# Lethality Validation for Human Pathogenic *Salmonella enterica* on Chicken Feathers and Blood during Simulated Commercial Low-Temperature Dry Rendering

**DOI:** 10.3390/microorganisms11082071

**Published:** 2023-08-12

**Authors:** Aime L. Shimwa Mvuyekure, Rosana G. Moreira, Thomas Matthew Taylor

**Affiliations:** 1 Department of Animal Science, Texas A&M University, College Station, TX 77843, USA; aime-leandre@tamu.edu; 2 Department of Biological and Agricultural Engineering, Texas A&M University, College Station, TX 77843, USA; rosana.moreira@ag.tamu.edu

**Keywords:** *Salmonella* inactivation, rendering, validation, poultry offal, blood meal, feather meal

## Abstract

Poultry rendering is the process of upcycling inedible poultry carcass materials into useful animal food/feed components as well as other valuable commercial products. Microbiological safety validation is nonetheless critical to ensuring the prevention of food safety hazard(s) transmission. This study determined the death kinetics of the thermotolerant *Salmonella enterica* serovar Senftenberg isolate 775W in chicken feathers and blood in low-temperature dry rendering (i.e., no direct contact with heating medium) to validate pathogen inactivation in commercial processing. Chicken feathers and blood were inoculated with *Salmonella* Senftenberg 775W and heated to 60, 70, or 80 °C for up to 60, 20, and 5 min, respectively. Three identically completed replicates (*N* = 3) for each product were conducted. Pathogen inactivation data were fitted to a non-linear model, providing for the detection and characterization of shoulder, log-linear death, and tailing components in death curves. The analysis showed a >7-log_10_ reduction in *Salmonella* was achieved across all processing temperatures, with *t*_7*D*_ values (time for 7.0 log-cycle lethality) ranging from 21.68, 7.30, and 4.26 min for feathers and 18.38, 5.03, and 2.79 min in blood at 60, 70, and 80 °C, respectively. Study findings validate that low-temperature processing conditions can inactivate *Salmonella* in poultry-rendered offal.

## 1. Introduction

Animal carcass rendering is a key prerequisite during companion animal food and livestock feedstock processing, as well as some biological soil amendment manufacturing (e.g., blood meal), recycling unused carcass materials into high-quality protein meals and useful products. Nearly 50% of animal carcasses are considered inedible, and if not further processed into useful products, their accumulation can create environmental hazards and pose risks to public health [1].

Rendering of poultry carcass offal must be monitored and validated to inactivate potential cross-contaminating animal and human pathogens such as *Campylobacter*, *Salmonella*, *Clostridium perfringens*, *Listeria monocytogenes*, and *Escherichia coli* [2]. High-temperature continuous rendering (115–145 °C) has been demonstrated to be lethal to microorganisms present in raw offal/carcass materials and sufficient to kill contaminating human and/or animal pathogenic bacteria [3,4,5]. Nevertheless, renderers may seek to render fats from carcass offal at lower temperatures in order to capture greater value during subsequent oleo-process applications such as soaps, candles, crayons, and various other consumer products [6]. The remaining high protein-yielding meals and other products from such lower temperature rendering processing, while not possessing equivalent financial value, are usefully converted into materials for manufacture into animal feeds as well as other products like meals (e.g., feather, blood meal) that can be used as biological soil amendments.

The U.S. Food and Drug Administration (FDA) Food Safety Modernization Act (FSMA) requires renderers to develop a hazard analysis plan and apply food safety preventive controls for hazards control. According to the Final Rule: Current Good Manufacturing Practice, Hazard Analysis, and Risk-Based Preventive Controls for Food for Animals (Title 21, U.S. Code of Federal Regulations §507), a process preventive control described within a facility’s food safety plan must be scientifically validated to control food safety hazards (biological, chemical, and/or physical). Validation methods are varied and may include scientific experimentation that demonstrates hazard(s) control under conditions identical to or sufficiently mimicking a commercial process [7,8]. Pandey et al. [9] reported that, during *Salmonella* Typhimurium inactivation in ground poultry carcasses, *Salmonella* could survive up to 120 min at a low rendering temperature (70 °C), wherein the pathogen was inoculated but not continuously agitated in the carcass material. Wong de la Rosa et al. [10], however, reported a similar trend where *D*-values of *Salmonella* Senftenberg 775W inoculated in whole chicken blood decreased from 0.61 to 0.12 min with an increase in processing temperature from 82.2 to 93.3 °C, with an eventual loss of detection by plating at higher temperatures. Similarly, Ramirez-Hernandez et al. [11] reported that the *D*-value decreased from 2.18 to 0.20 min as the temperature increased from 60 to 95 °C for low-fat beef rendered products and from 0.28 to 0.20 min for 50% fat beef offal.

There is a general lack of *Salmonella* lethality validation available to commercial renderers for use in food safety protection verification specific to chicken offal components during low-temperature rendering with the intent of producing feather or blood meal. Consequently, this study was conducted to determine the death kinetics of a thermo-tolerant *Salmonella enterica* isolate (Senftenberg 775W) in chicken feathers and blood in simulated commercial low-temperature dry rendering conditions (i.e., no direct contact between sample tissue and heat source). Targeted heating temperatures for feather and blood rendering validation were selected in consultation with industry experts in food safety validation for animal offal rendering (Pond, A. 2021. Personal communication.) The application of predictive mathematical modeling processes to microbiological data was thereafter conducted to provide further validation to commercial low-temperature rendering processes for pathogen control and food safety protection.

## 2. Materials and Methods

### 2.1. Microorganism Preparation

An isolate of *Salmonella enterica* serovar Senftenberg 775W from the American Type Culture Collection (ATCC 43845, Manassas, VA, USA) was revived from −80 °C cryo-storage from the Food Microbiology Laboratory culture collection (Department of Animal Science, Texas A&M University, College Station, TX, USA). This isolate was selected based on its substantially higher heat resistance to simulated poultry offal rendering previously observed by our research group as well as its belonging to the serovar previously reported as the most frequently recovered *Salmonella enterica* serovar from commercially rendered animal-derived products [4,12]. The organism was revived by first passing frozen culture into 10.0 mL of sterile tryptic soy broth (TSB; Becton, Dickinson and Co., Sparks, MD, USA) and then incubating for 24 h at 37 °C. Thereafter, the organism was aseptically passed into a new sterile volume of TSB and again incubated at 37 °C for a 24 h period. The isolate was then streaked for isolation onto tryptic soy agar (TSA; Becton, Dickinson and Co.), and an isolated colony was picked and prepared for serotyping to verify the organism’s identity and serovar. The *Salmonella* isolate was prepared and shipped for serotyping to the National Veterinary Services Laboratory (NVSL) (U.S. Department of Agriculture-Agricultural Research Service, Ames, IA, USA). Using previously published methods, a mutant organism spontaneously resistant to 100.0 μg/mL rifampicin (Rif^+^) was prepared to facilitate selective recovery of the organism from inoculated feathers and blood without potential complications caused by any background *Salmonella* enterica [13,14]. To ensure antibiotic resistance development did not produce a significant difference in the resulting mutant organism’s heat tolerance, parent and mutant isolates of *S*. Senftenberg were compared for growth kinetics in a liquid microbiological medium and for heat resistance at 60 °C prior to the initiation of inoculated chicken feather and blood experimentation.

### 2.2. Salmonella Parent and Rif^+^ Isolates Growth Comparison

Growth and replication capabilities of the parent and mutant (Rif^+^) *S*. Senftenberg organisms were compared by first diluting pure culture in TSB to a target of 10^2^ CFU/mL to ensure Rif^+^ status did not impede cell growth and prediction of its entry into the early stationary phase for later experimental work. Vials containing the parent or mutant organism were incubated at 37 °C and then removed after 0, 1, 2, 4, 6, 8, 10, 12, 14, and 18 h to compare population counts and determine each’s entry into the early stationary phase (expected to produce the greatest heartiness to the application of heat). Parent cells of *Salmonella* Senftenberg were enumerated on Aerobic Count Plate (ACP) Petrifilms™ (3M™, Minneapolis, MN, USA) and Rif^+^ *Salmonella* cells on TSA supplemented with 100.0 μg/mL rifampicin (Sigma-Aldrich Co., St. Louis, MO, USA; TSAR). Following incubation of plates at 37 °C for 36 h, colonies were counted, and the resulting data were log_10_-transformed for subsequent analysis.

### 2.3. In Vitro Thermal Death Time Comparison for S. Senftenberg Parent and Rif^+^ Isolates

A preliminary study was conducted to verify that rifampicin resistance did not significantly affect the heat resistance of the Rif^+^ *S*. Senftenberg isolate versus its parent. Inocula for both parent and Rif^+^ isolates were prepared using the procedure described in Section 2.2 with 15 mL of sterile Phosphate Buffered Saline (PBS; Thermo-Fisher Science, Waltham, MA, USA) instead of TSB for inoculum preparation. To obtain an inoculum, 15 mL of PBS was added to the pellet and vortexed for 1 min. After inoculum preparation, 0.5 mL of an inoculum (parent or Rif^+^) was aseptically pipetted into disposable borosilicate glass culture tubes (6 × 50 mm; VWR Int., Radnor, PA, USA) for thermal processing. Culture-loaded tubes were submerged in a water bath tempered to 60 °C. To monitor temperature change and come-up time, a Type-K thermocouple was inserted in a control culture tube filled with 0.5 mL of the PBS diluent but not containing any *Salmonella* cells.

Once the temperature inside the tubes reached 60 °C, culture tubes loaded with either *Salmonella* parent or Rif^+^ were removed after heating for 0, 0.5, 1, 1.5, 2, 3, 4, 5, 7.5, 10, 12, 15, 30, or 60 min. The removed culture tubes were placed immediately into an ice-cold water bath to prevent further bacterial lethality from residual heat. After 1 min, experimental vials were aseptically placed in sterile 50 mL conical plastic centrifuge tubes pre-loaded with 4.5 mL of sterile 0.1% peptone water diluent, and sterilized pestles were used to grind the heated tubes to completely disperse surviving *Salmonella* cells into the diluent. Further serial dilutions were then prepared in sterile 0.1% peptone diluent, and surviving cells were plated on ACP Petrifilms. Inoculated Petrifilms were incubated according to manufacturer guidance prior to colony enumeration, and the resulting data were log_10_-transformed prior to statistical analysis.

### 2.4. Chicken Blood and Feathers Inoculation with Salmonella and Preparation for Thermal Lethality Testing

Whole chicken blood and raw chicken feathers were collected from a Texas-located commercial independent renderer in pre-sterilized containers and returned immediately to the Food Microbiology Laboratory at Texas A&M University. Upon return to the laboratory, aliquots of blood or feathers were prepared for immediate use or refrigerated (5 °C) until needed for experimentation. For chicken feathers, samples of 10 g each were weighed into a 50 mL sterile conical-bottom centrifuge tube. The feathers were then inoculated with 0.1 mL of prepared inoculum (*S*. Senftenberg Rif^+^) to achieve a target inoculum of 8.0–9.0 log_10_ CFU/g and vortexed for 1 min to mix the inoculum homogeneously over the feathers. For chicken blood, 7.5 mL chicken blood aliquots were aseptically transferred into 15 mL sterile conical-bottom centrifuge tubes. Samples were inoculated with 0.1 mL of Rif^+^ *Salmonella* to achieve a target inoculum of 8.0–9.0 log_10_ CFU/mL and vortexed for 1 min to homogenize the inoculum in blood.

### 2.5. Sample Thermal Lethality Processing for Feathers and Blood Samples

To ensure homogenous heat penetration into sample-containing conical vials, a circulating water bath was first cleaned and disinfected using 70% (*v*/*v*) ethyl alcohol before being filled with distilled water and tuned to the necessary water temperatures to heat samples to targeted temperatures. An alcohol bulb thermometer and Type K thermocouple were used to monitor the temperature of the water and verify that the sample came up to the target temperature, respectively. To insulate against heat loss, the water bath was covered with aluminum foil. The water bath was left covered for the entire processing time before taking out samples to minimize heat loss. Samples of Rif^+^ *Salmonella* Senftenberg heating periods (post-come-up) ranged from 0 to 60 min at 60 °C, 0 to 20 min at 70 °C, and 0 to 5 min at 80 °C. Samples were collected in time increments selected to be appropriate for the total processing time for each heating temperature. Following heating, samples were removed from heat and placed in ice-cold water for 1.0 min to stop any residual heating-derived lethality, and then surviving *S*. Senftenberg cells were enumerated on TSAR as described below (Section 2.6).

For inoculated chicken blood, inoculated samples in 15 mL conical-bottom centrifuge tubes were processed similarly to feathers. The processing times post-come-up ranged from 0 to 60 min at 60 °C, 0 to 10 min at 70 °C, and 0 to 5 min at 80 °C. Samples were incrementally sampled in a similar fashion to experiments testing lethality on feathers. To hold the centrifuge tubes submerged in the water, a tray was placed on top of the tubes and held them submerged horizontally for the entire processing time for both feathers and blood. Like feathers, samples were placed in an ice-cold water bath immediately following processing to prevent further thermal lethality of the isolates. Surviving *S*. Senftenberg Rif^+^ cells were then enumerated on TSAR for microbial survival determination (Section 2.6).

### 2.6. Salmonella Senftenberg Rif^+^ Microbiological Enumeration

For feather samples, treated samples were transferred aseptically into a sterile stomacher bag post cooling in an ice water bath using flame-sterilized tools and diluted with 90.0 mL of sterile 0.1% peptone diluent. Samples were stomached for 30 s at 300 rpm to homogenize the surviving microorganisms. Serial dilutions were prepared in a sterile 0.1% peptone diluent and enumerated on TSAR plates. The plates were incubated for 24–36 h at 37 °C before colony counting. For blood, after thermal treatment and cooling in an ice bath, the blood appeared fully coagulated; samples were transferred aseptically using flame-sterilized tools into sterile stomach bags and diluted with sterile 0.1% peptone diluent to achieve an appropriate decimal dilution. Samples were then further diluted in sterile 0.1% peptone diluent and plated on TSAR plates to enumerate surviving Rif^+^ *S*. Senftenberg. The plates were incubated for 24 h at 37 °C before colony counting. Resulting plate counts were log10-transformed prior to statistical analysis; the limit of detection of plating was 1.0 log_10_ CFU/g of feathers or blood.

### 2.7. Predictive Model Development

Preliminary growth comparison and *D*_60°C_ comparison experiments were designed as complete block experiments and replicated three times (*N* = 3). *Salmonella* growth data were fitted to the Baranyi and Roberts [15] model using the online curve fitting tool DMFit within the ComBase online predictive microbiology database (https://www.combase.cc/index.php/en/, accessed 21 July 2023). Curve fitting parameters were determined for each organism and each replicate and then subjected to a two-way unpaired *t*-test with Welch’s correction to identify differences in model-predicted values for *N*_0_ (initial count of organisms; log_10_ CFU/mL), *N*_fin_ (final count of organisms; log_10_ CFU/mL), lag phase duration (h), and μ_max_ (max. specific growth rate; 1/h) between the parent and Rif^+^ *S*. Senftenberg isolates. Means were considered different from one another at *p* < 0.05 and were compared using Prism v9.5 (GraphPad Software, LLC, San Diego, CA, USA).

In order to determine the *D*-value for each organism, components of log-linear death curves showing good linearity were selected and clipped from other data [4]. Thereafter, linear regression analysis was completed using the toolkit in Microsoft Excel for Macintosh (Office 365 v16, Redmond, WA, USA). The slope of the line was recorded for each replicate for each organism, and the negative inverse of the slope was calculated as the *D*_60°C_. Mean *D*_60°C_ values across three replicates for *Salmonella* parent and Rif^+^ were compared by unpaired two-tailed *t*-test (*p* < 0.05) with Welch’s correction to determine if the parent and mutant differed with respect to heat sensitivity. Data analysis was completed using Prism v.9.5 (GraphPad Software, LLC).

Experiments involving *Salmonella*-inoculated feathers or blood were designed as complete block-type experiments, completing three replicates (*N* = 3). The Microsoft Excel Add-In software package GInaFiT was utilized to apply curve fitting/empirical model fitting to log-transformed *S*. Senftenberg Rif^+^ count data to identify the optimal primary model for predicting lethality during low-temperature rendering of chicken feathers and blood [16]. Following the identification of the optimal model (e.g., Geeraerd model, Baranyi and Roberts, Weibull, etc.) model parameters of shoulder length (*S*_L_), defined as the initial non-changing count in the microbial population that results as cells accumulate heat to lethal levels prior to exhibiting exponential declines in populations, *k*_max_ (maximum rate of decline of organisms observed during model-predicted lethality, and *N*_res_ (minimum number of surviving cells of the pathogen predicted by the model), were generated and compared to observed values for these parameters in order to determine which model type best fit the experimental data for each sample type over the three processing temperatures. For model fitting/goodness of fit estimation, the model coefficient of determination (R^2^) and standard error of fitting were determined and used to indicate which exhibited the best degree of data fitting/predictive value.

The two models showing the highest degrees of goodness of fit to experimental data were the thermal inactivation models by Geeraerd et al. [17] and Baranyi et al. [18]. Both can describe shoulders and/or tails as well as the log-linear decrease of a microbial population as a function of heating for a particular period of time. The Geeraerd model can accommodate log-linear behavior with and without shoulder and/or tailing, revealing a smooth transition between each phase. For this model, tailing is considered for a population remaining constant in time or, otherwise stated, not undergoing any significant subsequent inactivation [17]. In their study, these authors proposed how the parameters for model fitting using this approach relate to each other as expressed in Equation (1).

(1) $$N\left(t\right)=\left(N_{o}-N_{res}\right)e^{-k_{max}t}\left(\frac{e^{k_{max}S_{L}}}{1+(e^{k_{max}S_{L}}-1)e^{-k_{max}t}}\right)+N_{res}$$

where *N*_0_ is the initial microbial population, *k_max_* is the specific inactivation rate, *N_res_* is the residual microbial population, *S_L_* is the shoulder length, and *t* is time. Baranyi et al. [18] considered inactivation curves more like the mirror image of a growth curve. Their model fitting approaches the inactivation curves as sigmoidal survival curves with shoulder and tail/or tail adjustment functions and a log-linear decrease of the microbial population. Like Geeraerd’s team, Baranyi et al. [18] proposed how the parameters for model fitting using this approach relate to each other, as expressed in Equation (2).

(2) $$N\left(t\right)=\left(N_{o}-N_{min}\right)e^{-k_{max}(t-B\left(t\right))}+N_{min}$$

where *N*_0_ is the initial microbial population, r is shoulder length, *k_max_* is the specific inactivation, *N_min_* is the minimum cell concentration remaining in the tailing phase, *B(t)* is the shoulder function that relates to time (*t*), and shoulder length (*r*) and is given by Equation (3) below.

(3) $$B\left(t\right)=\frac{r}{3}\left(\frac{1}{2}ln\frac{{\left(r+t\right)}^{2}}{r^{2}-rt+t^{2}}+\sqrt{3}arctan\frac{2t-r}{r\sqrt{3}}+\sqrt{3}arctan\frac{1}{\sqrt{3}}\right)$$

Previous research has defended the use of death kinetic parameters such as the *t*_4*D*_ (time to achieve a 4.0 log_10_-cycle inactivation; analogous to the F-value principle) as an alternative to the more traditional *D*-value [19,20]. This has been particularly useful when microbial death curves do not follow a strictly log-linear trend throughout the entire heating period. As proposed by Whiting [21], *t*_4*D*_ accommodates the presence of a shoulder as well as a log-linear decline in bacterial population during thermal inactivation trials (Equation (4)).

(4) $$t_{4D}=S_{L}+4D$$

### 2.8. Statistical Analysis of Data

Statistically significant differences as a function of processing temperature for model parameters were determined by one-way analysis of variance (AOV); differing means were separated by Fisher’s Least Squares Differences (LSD) test at *p* < 0.05. The statistical analysis of the data was completed using the general linear model within JMP v.16 (SAS Institute, Inc., Cary, NC, USA).

## 3. Results and Discussion

### 3.1. In Vitro Growth of Salmonella Senftenberg 77W Parent and Rif^+^ Organisms

**Figure 1:**
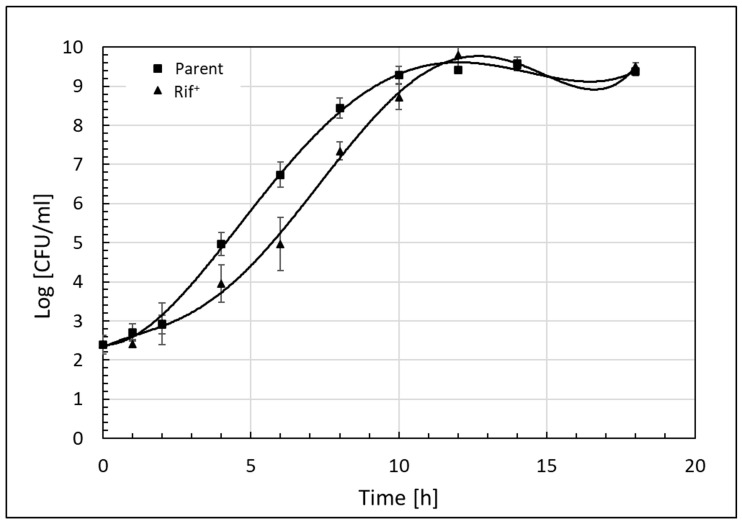
Growth of *Salmonella* Senftenberg 775W in tryptic soy broth at 37 °C. Symbols depict least squares means from three replications (*N* = 3); error bars depict the standard deviation of the mean.

Figure 1 shows the parent *Salmonella* Senftenberg exhibited quicker entry into logarithmic growth versus the Rif^+^ mutant, with a significantly shorter lag period versus the drug-resistant mutant (*p* = 0.024; Table 1).

Nevertheless, the Rif^+^ mutant accelerated growth during the logarithmic phase, achieving non-statistically differing counts versus the parent isolate at 10 h and thereafter (*p* = 0.069). The *t*-test analysis for μ_max_ (1/h) showed no significant difference in maximum specific growth rates of both organisms during the exponential phase (*p* = 0.422), providing validation that Rif^+^ mutant organism growth did not differ from that of the parent. This result is consistent with previous reports on this *S*. Senftenberg strain, indicating Rif^+^ capacity does not compromise growth substantially versus its parent [14].

### 3.2. D_60°C_ Value Comparison of Salmonella Senftenberg Parent and Rif^+^ Isolates

Subsequent to the completion of the growth curves comparison, a thermal inactivation study was carried out comparing the survival of *Salmonella* Senftenberg parent and mutant in PBS when heated at 60 °C, similar to studies previously conducted by our laboratory [4]. The average *D*_60°C_ of the *Salmonella* Senftenberg parent and mutant were 0.83 ± 0.24 and 1.12 ± 0.50 min, respectively. The antibiotic resistance appeared to produce a higher degree of heat resistance, though a two-tailed *t*-test indicated mean *D*-values did not statistically differ (*p* = 0.351). Cuervo et al. [14] conducted similar experiments on the same *S*. Senftenberg 775W isolate used in the current study and also reported no statistically significant differences in heat resistance between the parent and drug-resistant mutant. Hence, it was deemed appropriate for subsequent studies to include the use of Rif^+^ *S*. Senftenberg to facilitate the selective recovery of surviving cells.

### 3.3. Modeled Predicted Lethality of Salmonella on Chicken Feathers as a Function of Heating Temperature and Time

The Geeraerd and the Baranyi inactivation models both exhibited high degrees of data fitting when applied to the experimental log_10_-transformed data for the thermal inactivation of *Salmonella* on chicken feathers. The Geeraerd model showed improved goodness of fit (i.e., lower standard error and higher R^2^) (Table 2), making it optimal for data fitting for *Salmonella* inactivation at all three experimental processing temperatures [22].

**Figure 2:**
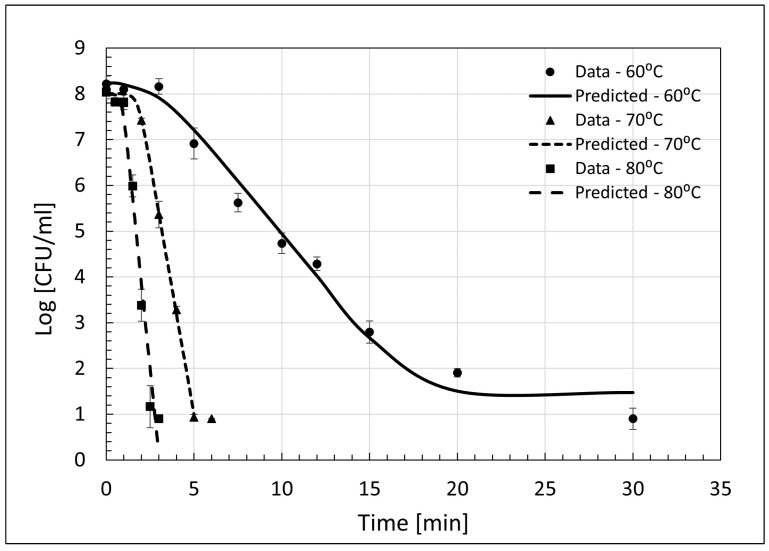
Survival of rifampicin-resistant *Salmonella* Senftenberg 775W inoculated on chicken feathers predicted by Geeraerd et al.’s [17] model at temperatures 60, 70, and 80 °C. Symbols depict the means of three identical complete replicates (*N* = 3); error bars depict one sample standard deviation from the mean. Surviving *Salmonella* were enumerated on tryptic soy agar supplemented with 100 μg/mL rifampicin following 24 h of incubation at 37 °C.

At all three processing temperatures, the existence of a shoulder, which represents an initial period of non-decline during which *Salmonella* cells absorb heat to a fatal degree before die-off, was detected [23]. As was expected, the shoulder length was significantly different across all three processing temperatures (*p* = 0.009), and the duration of the shoulder declined from approximately 6.1 min at 60 °C to approximately 2.4 min at 80 °C. Model-predicted initial *Salmonella* counts (*N*_0_) did not differ from one another (*p* = 0.492), and *N*_res_ populations at 60 and 70 °C were predicted below the limit of detection of the plating assay for feathers (1.0 log CFU/g). Hence, these results do not sufficiently validate the utility of either primary model to achieve more than 7.0 log_10_-cycle reductions in the *Salmonella* population. Nonetheless, the Geeraerd model demonstrated the simulated rendering processes effectively reduced *Salmonella* to non-detectable counts on rendered chicken feathers (Figure 2). In the current study, at all temperatures for feathers, there was increased variability in plate counts at intermediate time points during heating as compared to blood. Given the high consistency of inoculation of feathers (Figure 2) at all temperatures, it is likely that despite efforts to ensure efficient homogenous heating by submerging samples in a water bath, air pockets in between individual feathers produced an insulating effect on individual cells in samples across the replicates. This might be comparable to commercial rendering, where, despite differences in scale, feathers not tightly packed together during processing would be expected to be non-homogenously heated at variable times during processing.

Our team previously used it to describe the lethality of *S*. Senftenberg in chicken blood heated at higher processing temperatures (82–93 °C) [10]. In the current study, the calculated *t*_4*D*_ periods for 60, 70, and 80 °C using the Geeraerd model were 14.60 ± 0.46, 5.40 ± 0.70, and 3.37 ± 0.20 min, respectively. The value of a single *D* when the microbial populations are in log-linear decline (i.e., not in the shoulder phase) can be calculated as the difference of the *t*_4*D*_ minus the corresponding *S*_L_ and divided by 4.0 [10]. The calculated *D*-values for *S*. Senftenberg 775W used on feathers at the three experimental temperatures from the current study using the data in Table 2 above were 2.23, 0.66, and 0.29 min at 60, 70, and 80 °C, respectively. By extension, the *t*_4*D*_ can be used to extrapolate a *t*_7*D*_ (time necessary to achieve a 7.0 log_10_-cycle reduction) by multiplying the temperature-specific *D*-value by 7.0 and summing the product with the appropriate *S*_L_. In the current study, this would yield *t*_7*D*_ values in feathers of 21.68, 7.30, and 4.26 min at 60, 70, and 80 °C, respectively. This *t*_7*D*_ value accords a level of lethality comparable to the minimum *Salmonella* lethality requirement for fully cooked poultry products as imposed by the USDA’s Food Safety and Inspection Service [24].

### 3.4. Predicting Lethality to Salmonella in Chicken Blood

Similar to observations for feathers, the Geeraerd et al. [17] model effectively fits chicken blood *Salmonella* survival experimental data at each of the heating temperatures. Table 3 presents model parameters for chicken blood at differing heating temperatures. To that end, *D*-values were again calculated using the Geeraerd model-provided shoulder length (*S*_L_) by re-arranging the calculation for the *t*_4*D*_ as indicated above (Section 3.3).

**Figure 3:**
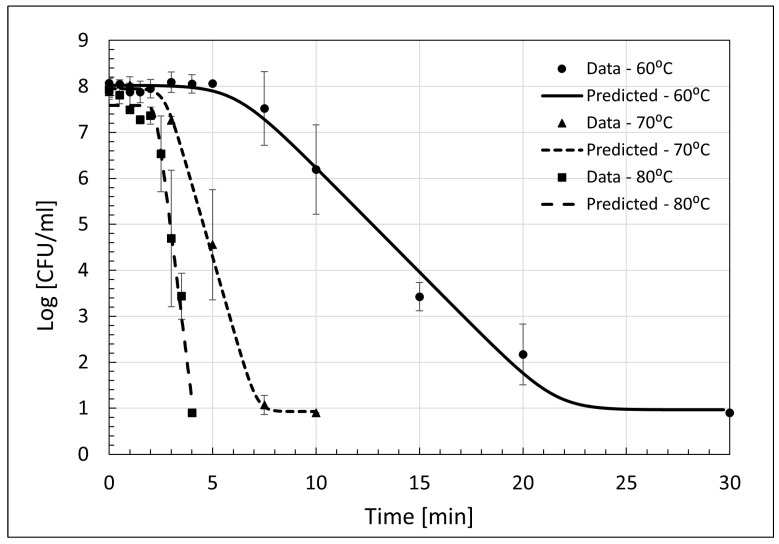
Survival of rifampicin-resistant *Salmonella* Senftenberg 775W inoculated on chicken blood predicted by Geeraerd et al.’s [17] model at temperatures 60, 70, and 80 °C. Symbols depict the means of three identical complete replicates (*N* = 3); error bars depict one sample standard deviation from the mean. Surviving *Salmonella* were enumerated on tryptic soy agar supplemented with 100 μg/mL rifampicin following 24 h of incubation at 37 °C.

Unlike for feathers, *Salmonella* lethality curves produced by model-fitting the chicken blood experimental data did not present tail components at 70 and 80 °C (Figure 3). These demonstrated no apparent continued inactivation of the pathogen following a steep exponential decline in microbe numbers at 70 and 80 °C, as plate counts fell below the limit of detection. Shoulders were detected in the data, though in blood they were much shorter than in feathers. This may have occurred due to the initial liquid nature of the blood and the more effective homogenization of heating versus feathers due to less air in the sample vial and the homogenous mixing of *Salmonella* cells throughout the blood. The presence of cold spots and non-homogenous heat distribution during processing has been reported as a contributing factor to the higher numbers observed in solid food products that cannot evenly distribute heat [4,25]. In addition, these results were not entirely unexpected given the changes in moisture content of feathers and blood occurring during processing. Doyle and Mazzota [26] reported in their review that the thermal resistance of *Salmonella* increases in low-water activity products, presumably due to less efficiency in heat transfer from the surrounding material to the pathogen.

As was the case with chicken feathers, the *S*_L_ was inversely correlated with processing temperature. As the processing temperature was increased from 60 to 80 °C, the time needed for cells to initially accumulate sufficient heat to exhibit death decreased in a predictable fashion, though statistical differences in mean *S*_L_ values were most clearly observed between the lowest and highest processing temperatures. Likewise, the *t*_4*D*_ for the pathogen at each of the three processing temperatures also decreased with increasing processing temperature; *t*_4*D*_ values for the *S*. Senftenberg Rif^+^ at 60, 70, and 80 °C were 11.8 ± 0.17, 3.69 ± 0.04, and 1.92 ± 0.03 min, respectively. *D*-values derived from these, accounting for the presence and durations of shoulders at these three temperatures (Table 3), were 2.22, 0.46, and 0.27 min at 60, 70, and 80 °C, respectively. The resulting calculated *t*_7*D*_ values at 60, 70, and 80 °C were therefore 18.38, 5.03, and 2.79 min. This relationship between temperature and *Salmonella D*-values was also reported by Wong de la Rosa et al. [10] and applied to higher processing temperatures for chicken blood. For both chicken feathers and blood, the relationship between temperature and shoulder parameters was determined to be linear and decrease with an increase in temperature.

### 3.5. Secondary Modeling Validation of Salmonella Lethality during Simulated Commercial Rendering

Equation (5) is the square root model of Ratkowsky et al. [27] used to model the effect of temperature on the *k*_max_ parameter, where *T_min_* is the theoretical minimum temperature for inactivation. By fitting the experimental data in the secondary model Equation (5), the minimum theoretical temperature for which *Salmonella* Senftenberg can be inactivated in chicken feathers and blood was 49.23 and 48.28 °C, respectively.

(5) $$\sqrt{k_{max}}=b\times \left(T-T_{min}\right)$$

High-temperature processing presents the challenge of exposing animal protein and the resulting tallow to heating temperatures that reduce quality and utility in differing industries and introduce colors and odors that lower market prices for tallow and protein meals [5]. Since most components of rendered products used by pet and livestock food manufacturers are proteins and fats, low-temperature dry rendering could play an important role in the preservation of fat and protein quality compared to high-temperature rendering. Additionally, the use of lower temperatures for rendering animal offal opens the opportunity for enhanced value retention for fats through further oleochemical processing requiring softer fats or those retaining some unsaturation for subsequent processing into various products [1]. However, it is crucial that this process be validated to ensure that it does not compromise the microbiological safety of rendered products. The research experimental data showed that the total lethality of *Salmonella* inoculated on chicken blood was achieved after approximately 22, 4, and 2.5 min of heating at 60, 70, and 80 °C, respectively, and for inoculated chicken feathers after 22, 7.3, and 4.3 min of heating at 60, 70, and 80 °C, respectively. The results provide validation of effective pathogen inactivation and food safety control for commercial renderers using a low-temperature rendering process for the manufacture of chicken-derived blood and feather meals.

## 4. Conclusions

This research provides baseline information on the validation of *Salmonella* inactivation during the rendering of chicken feathers, complements previous research about inactivation in chicken blood, and lays the foundation for potential future research on poultry-rendered materials. However, similar questions should be asked to provide validation for the process used in rendering other non-poultry inedible and waste materials that are processed by renderers. According to our knowledge, there are only a few data points characterizing pathogen inactivation in cattle and swine-rendered materials, which should also be studied to provide food safety compliance assistance to the rendering industries in the U.S. and countries importing rendered materials. Future studies are recommended focusing on identifying and validating the usefulness of pathogen surrogate microbes or other materials that may facilitate in-plant challenge trials to give more robust food safety hazards control validation for the rendering industry.

## Figures and Tables

**Table 1:** Parameters determined by fitting Baranyi and Roberts [15] complete growth model to parent and Rif^+^ *Salmonella* Senftenberg 775W growth in tryptic soy broth at 37 °C.

Model Parameter ^1^	*Salmonella* Parent	*Salmonella* Rif^+^	*p* < 0.05
*N*_0_ (log_10_ CFU/mL)	2.37 ± 0.26 ^2^	2.50 ± 0.08	0.453
Lag (h)	1.53 ± 0.23 A	2.99 ± 0.67 B	0.024
μ (1/h)	0.98 ± 0.03	0.94 ± 0.07	0.422
*N*_fin_ (log_10_ CFU/mL)	9.44 ± 0.10	9.63 ± 0.09	0.069
R^2^	0.997	0.986	0.231
Std. Error	0.153	0.357	0.148

^1^ *N*_0_: initial predicted plate count; Lag: lag phase; μ: maximum specific growth rate; *N*_fin_: model-predicted terminal plate count. ^2^ Values depict means from three replicates (*N* = 3) ± one sample standard deviation. Letters differing between means within a row (A, B) indicate means differ at *p* < 0.05 as determined from a two-way unpaired *t*-test.

**Table 2:** Parameters determined by fitting Geeraerd et al. [17] to Rif^+^ *Salmonella* Senftenberg 775W inactivation on chicken feathers at differing temperatures.

Model Parameter ^1^	60 °C	70 °C	80 °C	*p* < 0.05
*N*_0_ (log_10_ CFU/g)	8.03 + 0.10 ^2^	7.95 + 0.12	7.59 + 0.14	0.492
*S*_L_ (min)	6.07 + 0.64 A	2.68 + 0.16 B	2.23 + 0.11 B	0.009
*k*_max_ (1/min)	1.05 + 0.07 A	3.63 + 0.20 B	8.37 + 0.67 C	<0.01
*N*_res_ (log_10_ CFU/g)	0.96 + 0.26	0.93 + 0.14	-- ^3^	0.906
R^2^	0.992	0.998	0.988	
Std. Error	0.067	0.030	0.098	

^1^ *N*_0_: initial predicted plate count; *S*_L_: shoulder length; *k*_max_: maximum log-linear inactivation rate; *N*_res_: model-predicted terminal plate count. ^2^ Values depict means from three identical replicates (*N* = 3) ± one sample standard deviation. Letters differing between means within a row (A, B, and C) indicate means differ at *p* < 0.05. ^3^ Predictive models failed to indicate the presence of a tail: shoulder + log-linear death only.

**Table 3:** Parameters determined by fitting Geeraerd et al. [17] to Rif^+^ *Salmonella* Senftenberg 775W inactivation on chicken blood at different temperatures.

Model Parameter ^1^	60 °C	70 °C	80 °C	*p* < 0.05
*N*_0_ (log CFU/mL)	8.24 ± 0.24 ^2^	8.01 ± 0.12	7.99 ± 0.47	0.636
*S*_L_ (min)	2.84 ± 0.97 A	1.81 ± 0.11 AB	0.90 ± 0.23 B	0.002
*k*_max_ (1/min)	1.06 ± 0.10 A	5.06 ± 0.20 B	8.63 ± 1.10 C	<0.01
*N*_res_ (log CFU/mL)	1.47 ± 0.24	-- ^3^		
R^2^	0.991	0.998	0.971	
Std. Error	0.109	0.284	0.437	

^1^ *N*_0_: initial predicted plate count; *S*_L_: shoulder length; *k*_max_: maximum log-linear inactivation rate; *N*_res_: model-predicted terminal plate count. ^2^ Values depict means from three identical replicates (*N* = 3) ± one sample standard deviation. Letters differing between means within a row (A, B, and C) indicate means differ at *p* < 0.05. ^3^ Predictive model failed to indicate the presence of a tail, exhibiting shoulder + log-linear death only.

## Data Availability

Restrictions apply to the availability of these data. Data are available from the Corresponding Author upon gaining approval from the Fats and Proteins Research Foundation.
